# Early dynamics of transmission and control of COVID-19: a mathematical modelling study

**DOI:** 10.1016/S1473-3099(20)30144-4

**Published:** 2020-05

**Authors:** Adam J Kucharski, Timothy W Russell, Charlie Diamond, Yang Liu, John Edmunds, Sebastian Funk, Rosalind M Eggo, Centre for Mathematical Modelling of Infectious Diseases COVID-19 working group, Fiona Sun, Mark Jit, James D Munday, Nicholas Davies, Amy Gimma, Kevin van Zandvoort, Hamish Gibbs, Joel Hellewell, Christopher I Jarvis, Sam Clifford, Billy J Quilty, Nikos I Bosse, Sam Abbott, Petra Klepac, Stefan Flasche

**Affiliations:** a Centre for Mathematical Modelling of Infectious Diseases, London School of Hygiene & Tropical Medicine, London, UK

## Abstract

**Background:**

An outbreak of severe acute respiratory syndrome coronavirus 2 (SARS-CoV-2) has led to 95 333 confirmed cases as of March 5, 2020. Understanding the early transmission dynamics of the infection and evaluating the effectiveness of control measures is crucial for assessing the potential for sustained transmission to occur in new areas. Combining a mathematical model of severe SARS-CoV-2 transmission with four datasets from within and outside Wuhan, we estimated how transmission in Wuhan varied between December, 2019, and February, 2020. We used these estimates to assess the potential for sustained human-to-human transmission to occur in locations outside Wuhan if cases were introduced.

**Methods:**

We combined a stochastic transmission model with data on cases of coronavirus disease 2019 (COVID-19) in Wuhan and international cases that originated in Wuhan to estimate how transmission had varied over time during January, 2020, and February, 2020. Based on these estimates, we then calculated the probability that newly introduced cases might generate outbreaks in other areas. To estimate the early dynamics of transmission in Wuhan, we fitted a stochastic transmission dynamic model to multiple publicly available datasets on cases in Wuhan and internationally exported cases from Wuhan. The four datasets we fitted to were: daily number of new internationally exported cases (or lack thereof), by date of onset, as of Jan 26, 2020; daily number of new cases in Wuhan with no market exposure, by date of onset, between Dec 1, 2019, and Jan 1, 2020; daily number of new cases in China, by date of onset, between Dec 29, 2019, and Jan 23, 2020; and proportion of infected passengers on evacuation flights between Jan 29, 2020, and Feb 4, 2020. We used an additional two datasets for comparison with model outputs: daily number of new exported cases from Wuhan (or lack thereof) in countries with high connectivity to Wuhan (ie, top 20 most at-risk countries), by date of confirmation, as of Feb 10, 2020; and data on new confirmed cases reported in Wuhan between Jan 16, 2020, and Feb 11, 2020.

**Findings:**

We estimated that the median daily reproduction number (*R*_t_) in Wuhan declined from 2·35 (95% CI 1·15–4·77) 1 week before travel restrictions were introduced on Jan 23, 2020, to 1·05 (0·41–2·39) 1 week after. Based on our estimates of *R*_t_, assuming SARS-like variation, we calculated that in locations with similar transmission potential to Wuhan in early January, once there are at least four independently introduced cases, there is a more than 50% chance the infection will establish within that population.

**Interpretation:**

Our results show that COVID-19 transmission probably declined in Wuhan during late January, 2020, coinciding with the introduction of travel control measures. As more cases arrive in international locations with similar transmission potential to Wuhan before these control measures, it is likely many chains of transmission will fail to establish initially, but might lead to new outbreaks eventually.

**Funding:**

Wellcome Trust, Health Data Research UK, Bill & Melinda Gates Foundation, and National Institute for Health Research.

## Introduction

As of Feb 13, 2020, an outbreak of coronavirus disease 2019 (COVID-19) has resulted in 46 997 confirmed cases.^1^ The outbreak was first identified in Wuhan, China, in December, 2019, with most early cases being reported in the city. Most internationally exported cases reported to date have history of travel to Wuhan.^2^ In the early stages of a new infectious disease outbreak, it is crucial to understand the transmission dynamics of the infection. Estimation of changes in transmission over time can provide insights into the epidemiological situation^3^ and identify whether outbreak control measures are having a measurable effect.^4^, ^5^ Such analysis can inform predictions about potential future growth,^6^ help estimate risk to other countries,^7^ and guide the design of alternative interventions.^8^

However, there are several challenges to such analyses, particularly in real time. There can be a delay to symptom appearance resulting from the incubation period and delay to confirmation of cases resulting from detection and testing capacity.^9^ Modelling approaches can account for such delays and uncertainty by explicitly incorporating delays resulting from the natural history of infection and reporting processes.^10^ Additionally, individual data sources might be biased, incomplete, or only capture certain aspects of the outbreak dynamics. Evidence synthesis approaches, which fit to multiple data sources rather than a single dataset (or datapoint) can enable more robust estimation of the underlying dynamics of transmission from noisy data.^11^, ^12^ Combining a mathematical model of severe acute respiratory syndrome coronavirus 2 (SARS-CoV-2) transmission with four datasets from within and outside Wuhan, we estimated how transmission in Wuhan varied between December, 2019, and February, 2020. We used these estimates to assess the potential for sustained human-to-human transmission to occur in locations outside Wuhan if cases were introduced.

**Research in context**

**Evidence before this study**

We searched PubMed, BioRxiv, and MedRxiv for articles published in English from inception to Feb 10, 2020, with the keywords “2019-nCoV”, “novel coronavirus”, “COVID-19”, “SARS-CoV-2” AND “reproduction number”, “R0”, “transmission”. We found several estimates of the basic reproduction number (R_0_) of severe acute respiratory syndrome coronavirus 2 (SARS-CoV-2), including average exponential growth rate estimates based on inferred or observed cases at a specific timepoint and early growth of the outbreak in China. However, we identified no estimates of how R_0_ had changed in Wuhan since control measures were introduced in late January or estimates that jointly fitted data within Wuhan to international exported cases and evacuation flights.

**Added value of this study**

Our study combines available evidence from multiple data sources, reducing the dependency of our estimates on a single timepoint or dataset. We estimate how transmission has varied over time, identify a decline in the reproduction number in late January to almost 1, coinciding with the introduction of large scale control measures, and show the potential implications of estimated transmission for outbreak risk in new locations.

**Implications of all the available evidence**

Coronavirus disease 2019 is currently showing sustained transmission in China, creating a substantial risk of outbreaks in other countries. However, if SARS-CoV-2 has Middle East respiratory syndrome coronavirus-like or SARS-CoV-like variability in transmission at the individual level, multiple introductions might be required before an outbreak takes hold.

## Methods

### Data sources

To estimate the early dynamics of transmission in Wuhan, we fitted a stochastic transmission dynamic model^13^ to multiple publicly available datasets on cases in Wuhan and internationally exported cases from Wuhan. The four datasets we fitted to were: daily number of new internationally exported cases (or lack thereof), by date of onset, as of Jan 26, 2020; daily number of new cases in Wuhan with no market exposure, by date of onset, between Dec 1, 2019, and Jan 1, 2020; daily number of new cases in China, by date of onset, between Dec 29, 2019, and Jan 23, 2020; and proportion of infected passengers on evacuation flights between Jan 29, 2020, and Feb 4, 2020 (appendix p 3). We used an additional two datasets for comparison with model outputs: daily number of new exported cases from Wuhan (or lack thereof) in countries with high connectivity to Wuhan (ie, top 20 most at-risk countries), by date of confirmation, as of Feb 10, 2020; and data on new confirmed cases reported in Wuhan between Jan 16, 2020, and Feb 11, 2020 (appendix p 3).

### Procedures

**Figure 1:**
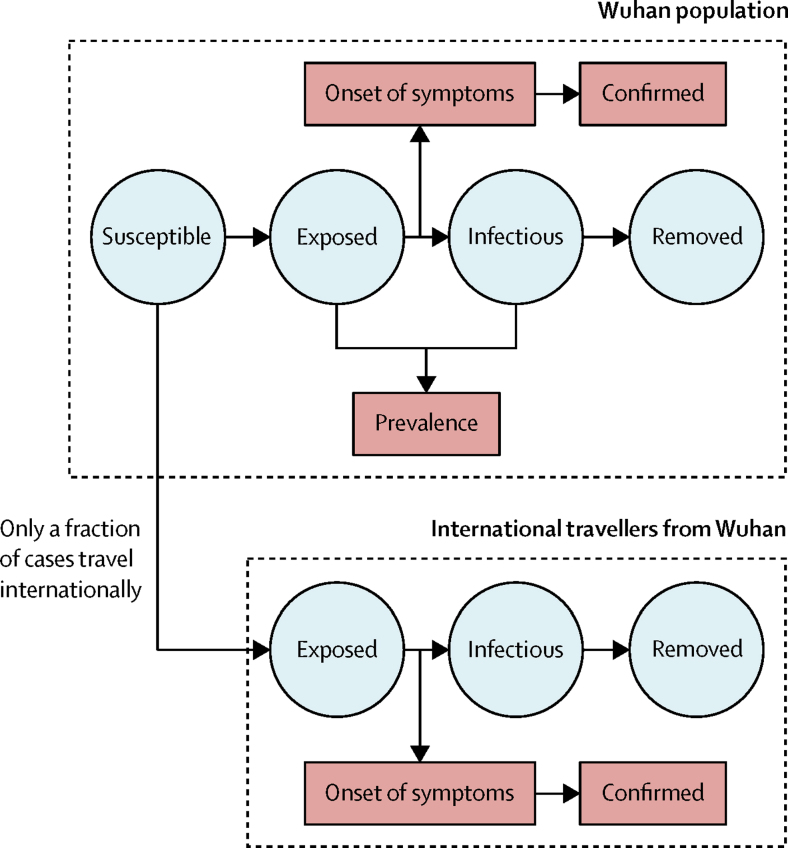
Model structure The population is divided into the following four classes: susceptible, exposed (and not yet symptomatic), infectious (and symptomatic), and removed (ie, isolated, recovered, or otherwise non-infectious). A fraction of exposed individuals subsequently travel and are eventually detected in their destination country.

In the model, we divided individuals into four infection classes, as follows: susceptible, exposed (but not yet infectious), infectious, and removed (ie, isolated, recovered, or otherwise no longer infectious; figure 1). The model accounted for delays in symptom onset and reporting by including compartments to reflect transitions between reporting states and disease states. The model also incorporated uncertainty in case observation, by explicitly modelling a Poisson observed process of newly symptomatic cases, reported onsets of new cases, reported confirmation of cases, and a binomial observation process for infection prevalence on evacuation flights (appendix pp 1–3). The incubation period was assumed to be Erlang distributed with mean 5·2 days^14^ (SD 3·7) and delay from onset to isolation was assumed to be Erlang distributed with mean 2·9 days (2·1).^2^, ^15^ The delay from onset to reporting was assumed to be exponentially distributed with mean 6·1 days (2·5).^2^ Once exposed to infection, a proportion of individuals travelled internationally and we assumed that the probability of cases being exported from Wuhan to a specific other country depended on the number of cases in Wuhan, the number of outbound travellers (assumed to be 3300 per day before travel restrictions were introduced on Jan 23, 2020, and zero after), the relative connectivity of different countries,^16^ and the relative probability of reporting a case outside Wuhan, to account for differences in clinical case definition, detection, and reporting within Wuhan and internationally. We considered the 20 countries outside China most at risk of exported cases in the analysis.

We modelled transmission as a geometric random walk process, and we used sequential Monte Carlo simulation to infer the transmission rate over time, as well as the resulting number of cases and the time-varying basic reproduction number (*R*_t_), defined here as the mean number of secondary cases generated by a typical infectious individual on each day in a full susceptible population. The model had three unknown parameters, which we estimated: magnitude of temporal variability in transmission, proportion of cases that would eventually be detectable, and relative probability of reporting a confirmed case within Wuhan compared with an internationally exported case that originated in Wuhan. We assumed the outbreak started with a single infectious case on Nov 22, 2019, and the entire population was initially susceptible. Once we had estimated *R*_t_, we used a branching process with a negative binomial offspring distribution to calculate the probability an introduced case would cause a large outbreak. We also did a sensitivity analysis on the following three key assumptions: we assumed the initial number of cases was ten rather than one; we assumed connectivity between countries followed WorldPop rather than MOBS Lab estimates; and we assumed that cases were infectious during the second half of their incubation period rather than only being infectious while symptomatic. All data and code required to reproduce the analysis is available online.

### Role of the funding source

The funder of the study had no role in study design, data collection, data analysis, data interpretation, or writing of the report. The corresponding author had full access to all the data in the study and had final responsibility for the decision to submit for publication.

## Results

**Figure 2:**
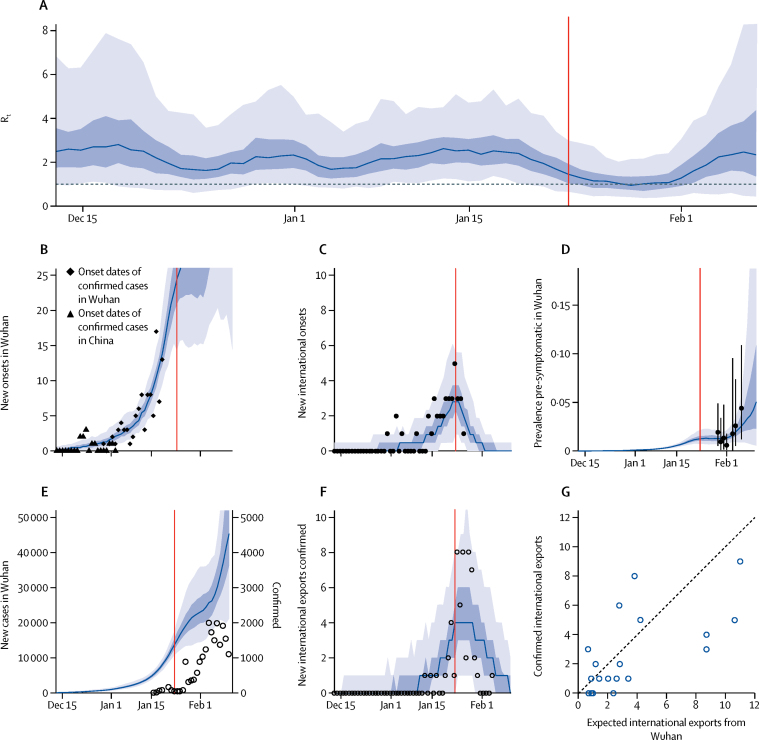
Dynamics of transmission in Wuhan, fitted up to Feb 11, 2020 The red line marks travel restrictions starting on Jan 23, 2020. For parts (A) to (F) blue lines represent median, light blue shading represents 50% confidence intervals of the model estimate, and dark blue shading represents 95% confidence intervals of the model estimate. In all panels, datasets that were fitted to are shown as solid points; non-fitted data are shown as empty circles. (A) Estimated *R*_t_ over time. The dashed line represents an *R*_t_ of 1. (B) Onset dates of confirmed cases in Wuhan and China. (C) Reported cases by date of onset (black points) and estimated internationally exported cases from Wuhan by date of onset (blue line). (D) Estimated prevalence of infections that did not have detectable symptoms (blue line), and proportion of passengers on evacuation flights that tested positive for severe acute respiratory syndrome coronavirus 2 (black points; error bars show 95% binomial CIs). (E) New confirmed cases by date in Wuhan (circles, right hand axis) and estimated new symptomatic cases (blue line, left hand axis). (F) International exportation events by date of confirmation of case, and expected number of exports in the fitted model. (G) Estimated number of internationally exported cases from Wuhan confirmed up to Feb 10, 2020 and observed number in 20 countries with the highest connectivity to China. *R*_t_=daily reproduction number.

We estimated that *R*_t_ varied during January, 2020, with median values ranging from 1·6 to 2·6 between Jan 1, 2020, and the introduction of travel restrictions on Jan 23, 2020 (figure 2). We estimated a decline in *R*_t_ in late January, from 2·35 (95% CI 1·15–4·77) on January 16, 1 week before the restrictions, to 1·05 (0·41–2·39) on January 31.

The model reproduced the observed temporal trend of cases within Wuhan and cases exported internationally. The model captured the exponential growth in case onsets in early January, the rising number of exported case onsets between Jan 15, and Jan 23, 2020, and the prevalence of infection measured on ten evacuation flights from Wuhan to seven countries. We estimated that 94·8% (95% CI 93·1–96·1%) of the Wuhan population were still susceptible on Jan 31, 2020 (figure 2). Our results suggested there were around ten times more symptomatic cases in Wuhan in late January than were reported as confirmed cases (figure 2), but the model did not predict the slowdown in cases that was observed in early February. The model could also reproduce the pattern of confirmed exported cases from Wuhan, which was not explicitly used in the model fitting (figure 2). We found that confirmed and estimated exported cases among the 20 countries most connected to China generally corresponded with each other, with the USA and Australia as notable outliers, having had more confirmed cases reported with a travel history to Wuhan than would be expected in the model (figure 2). There was evidence that the majority of cases were symptomatic. We estimated that 100% (95% CI 51–100) of cases would eventually have detectable symptoms, implying that most infections that were exported internationally from Wuhan in late January were in theory eventually detectable. As a sensitivity analysis, we repeated the analysis with a large number of initial cases, different mobility data, and the assumption that pre-symptomatic cases could transmit. In these analyses, we observed the same result of a decline in *R*_t_ from more than 2 to almost 1 in the last 2 weeks of January, 2020 (appendix pp 10–13).

**Figure 3:**
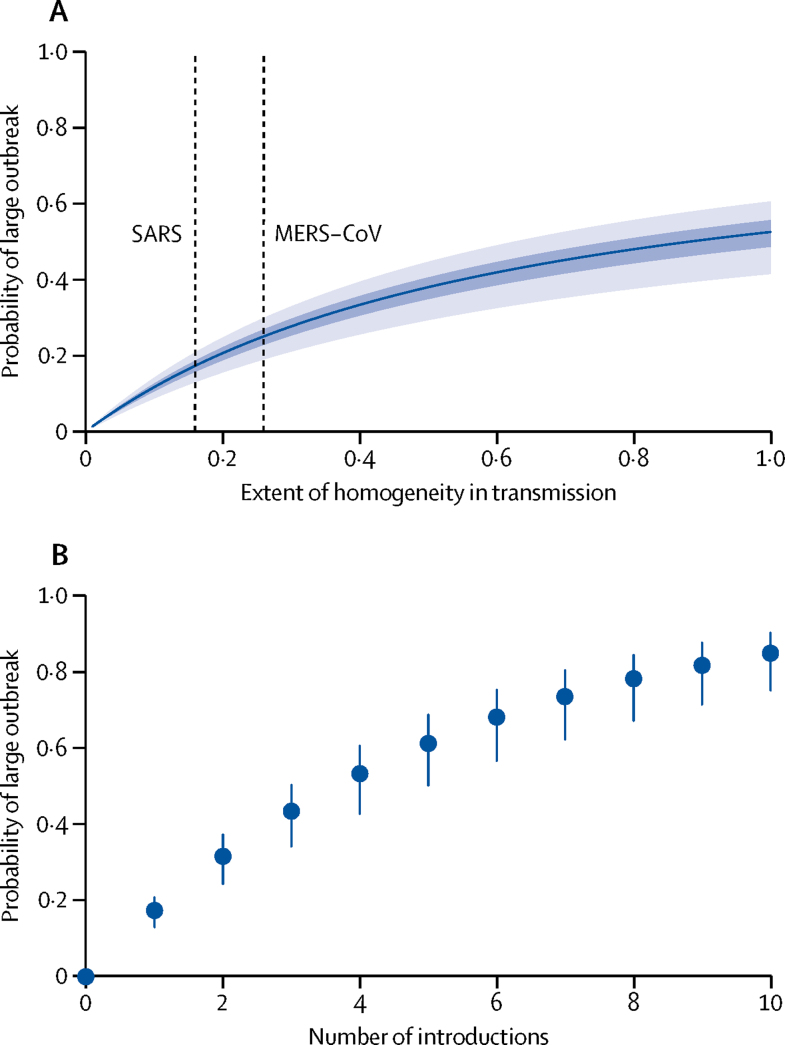
Risk that introduced infections will establish in a new population (A) Probability that a single case will lead to a large outbreak for different assumptions about the extent of homogeneity in individual-level transmission (ie, the dispersion parameter k in a negative binomial offspring process). Results are shown for the median reproduction number estimated for severe acute respiratory syndrome coronavirus 2 in Wuhan between Jan 1, 2020, and Jan 23, 2020. (B) Probability that a given number of introductions will result in a large outbreak, assuming SARS-like superspreading events can occur. Points show the median estimated reproduction number between Jan 1, 2020, and Jan 23, 2020; bars show 95% quantile of the range of median values of *R*_t_ during this period. SARS=severe acute respiratory syndrome. MERS=Middle East respiratory syndrome. *R*_t_=daily reproduction number.

To examine the potential for new outbreaks to establish in locations outside Wuhan, we used our estimates of the *R*_t_ to simulate new outbreaks with potential individual-level variation in transmission (ie, so called superspreading events).^17^, ^18^, ^19^ Such variation increases the fragility of transmission chains, making it less likely that an outbreak will take hold following a single introduction. If transmission is more homogeneous, with all infectious individuals generating a similar number of secondary cases, it is more likely than an outbreak will establish.^18^ Based on the median *R*_t_ estimated during January before travel restrictions were introduced, we estimated that a single introduction of SARS-CoV-2 with SARS-like or Middle East respiratory syndrome (MERS)-like individual-level variation in transmission would have a 17% to 25% probability of causing a large outbreak (figure 3). Assuming SARS-like variation and Wuhan-like transmission, we estimated that once four or more infections have been introduced into a new location, there is an over 50% chance that an outbreak will occur (figure 3).

## Discussion

Combining a mathematical model with multiple datasets, we found that the median daily *R*_t_ of SARS-CoV-2 in Wuhan probably varied between 1·6 and 2·6 in January, 2020, before travel restrictions were introduced. We also estimated that transmission declined by around half in the 2 weeks spanning the introduction of restrictions.

The estimated fluctuations in *R*_t_ were driven by the rise and fall in the number of cases, both in Wuhan and internationally, as well as prevalence on evacuation flights. Such fluctuations could be the result of changes in behaviour in the population at risk, or specific superspreading events that inflated the average estimate of transmission.^17^, ^18^, ^19^ We found some evidence of a reduction in *R*_t_ in the days before the introduction of travel restrictions in Wuhan, which might have reflected outbreak control efforts or growing awareness of SARS-CoV-2 during this period. The uncertainty in our estimates for *R*_t_ following the decline in early February, 2020, results from a paucity of data sources to inform changes in transmission during this period.

Comparing model predictions with observed confirmed cases reported in Wuhan, we found that the model predicted at least ten times higher cases than were reported in early February, 2020. The model also did not predict the more recent slowdown in cases, suggesting that transmission might have declined more than our model—which did not fit to this case data—estimated during early February, 2020. Our estimates for international cases in specific countries were broadly consistent with the number of subsequently confirmed exported cases outside Wuhan. However, there were notably more cases exported to France, USA, and Australia compared with what our model predicted. This could be the result of increased surveillance and detection as awareness of SARS-CoV-2 increased in late January, which would suggest earlier exported cases might have been missed, or could be the result of increased travel out of Wuhan immediately before introduction of travel restrictions on Jan 23, 2020.

Based our on estimated reproduction number and published estimates of individual-level variation in transmission for SARS-CoV and MERS-CoV, we found that a single case introduced to a new location would not necessarily lead to an outbreak. Even if the reproduction number is as high as in Wuhan in early January, it could take several introductions for an outbreak to establish, because high individual-level variation in transmission makes new chains of transmission more fragile, and hence it becomes less likely that a single infection will generate an outbreak. This factor highlights the importance of rapid case identification and subsequent isolation and other control measures to reduce the chance of onward chains of transmission.^20^

Our analysis highlights the value of combining multiple data sources in analysis of COVID-19. For example, the rapid growth of confirmed cases globally during late January, 2020, with case totals in some instances apparently doubling every day or so, would have had the effect of inflating *R*_t_ estimates to implausibly large values if only these recent datapoints were used in our analysis. Our results also have implications for estimation of transmission dynamics using the number of exported cases from a specific area.^21^ Once extensive travel restrictions are introduced, as they were in Wuhan, the signal from such data gets substantially weaker. If restrictions and subsequent delays in detection of cases are not accounted for, this could lead to artificially low estimates of *R*_t_ or inferred case totals from the apparently declining numbers of exported cases. Our model estimates benefited from the availability of testing data from evacuation flights, which allowed us to estimate current prevalence. Having such information for other settings, either through widespread testing or serological surveillance, will be valuable to reduce reliance on case reports alone.

There are several other limitations to our analysis. We used plausible biological parameters for SARS-CoV-2 based on current evidence, but these values might be refined as more comprehensive data become available. However, by fitting to multiple datasets to infer model parameters, and conducting sensitivity analyses on key areas of uncertainty, we have attempted to make the best possible use of the available evidence about SARS-CoV-2 transmission dynamics. Furthermore, we used publicly available connectivity and risk estimates based on international travel data to predict the number of cases exported into each country. These estimates have shown good correspondence with the distribution of exported cases to date,^22^ and are similar to another risk assessment for COVID-19 with different data.^23^ We also assumed that the latent period is equal to the incubation period (ie, individuals become infectious and symptomatic at the same time) and all infected individuals will eventually become symptomatic. However, there is evidence that transmission of SARS-CoV-2 can occur with few reported symptoms.^24^ Therefore, we did a sensitivity analysis in which transmission could occur in the second half of the incubation period, but this did not change our overall conclusions of a decline in *R*_t_ from around 2·4 to almost 1 during the last 2 weeks of January. We also explored having a larger initial spillover event and using different sources for flight connectivity data, both of which produced the same conclusion about the decline in transmission. In our analysis of new outbreaks, we also used estimates of individual-level variation in transmission for SARS and MERS-CoV to illustrate potential dynamics. However, it remains unclear what the precise extent of such variation is for SARS-CoV-2.^17^ If transmission were more homogenous than SARS-CoV or MERS-CoV, it would increase the risk of outbreaks following introduced cases. As more data become available, it will be possible to refine these estimates; therefore we have made an online tool so that users can explore these risk estimates if new data become available (appendix p 4).

Our results show that there was probably substantial variation in SARS-CoV-2 transmission over time, and suggest a decline in transmission in Wuhan in late January, 2020, around the time that control measures were introduced. If COVID-19 transmission is established outside Wuhan, understanding the effectiveness of control measures in different settings will be crucial for understanding the dynamics of the outbreak, and the likelihood that transmission can eventually be contained or effectively mitigated.

**Contributors**

Data analysis was led by AJK, who programmed the model with help from TWR. AJK, SF, and RME planned the inference framework. CD provided the data from online sources. The CMMID 2019-nCoV working group members contributed to processing, cleaning, and interpretation of data, interpreted the study findings, contributed to the manuscript, and approved the work for publication. All authors interpreted the findings, contributed to writing the manuscript, and approved the final version for publication.

**Declaration of interests**

We declare no competing interests.
