# Supplementary material for: Early dynamics of transmission and control of COVID-19: a mathematical modelling study
Source: Lancet Infect Dis. 2020 May;20(5):553–8. doi: 10.1016/S1473-3099(20)30144-4 (PMC7158569; doi:10.1016/S1473-3099(20)30144-4)
Supplement: Supplementary appendix [file mmc1.pdf]

THE LANCET
Infectious Diseases

Supplementary appendix

This appendix formed part of the original submission and has been peer reviewed. We post it as supplied by the authors.

This online publication has been corrected. The corrected version first appeared at thelancet.com on March 25, 2020.

Supplement to: Kucharski AJ, Russell TW, Diamond C, et al. Early dynamics of transmission and control of COVID-19: a mathematical modelling study. *Lancet Infect Dis* 2020; published online March 11. http://dx.doi.org/10.1016/S1473-3099(20)30144-4.

Appendix for Early dynamics of transmission and control of COVID-19: a mathematical modelling study

Adam J Kucharski[1], Timothy W Russell[1], Charlie Diamond[1], Yang Liu[1], CMMID COVID-19 working group, John Edmunds[1], Sebastian Funk[1], Rosalind M Eggo[1]*

[1]Centre for Mathematical Modelling of Infectious Diseases, London School of Hygiene & Tropical Medicine, London.

**The following authors were part of the CMMID nCoV working group: Fiona Sun, Mark Jit, James D Munday, Nicholas Davies, Amy Gimma, Kevin van Zandvoort, Hamish Gibbs, Joel Hellewell, Christopher I Jarvis, Sam Clifford, Billy J Quilty, Nikos I Bosse, Sam Abbott, Petra Klepac and Stefan Flasche.*

Supplementary methods

Transmission model structure

We formulated an extended version of an SEIR model to reflect the disease-specific dynamics. The model extensions include: a class for asymptomatic cases, two cumulative classes - one for symptomatic cases and the other for confirmed cases – and two identical classes for exposed and infected individuals resulting in Erlang distributed incubation and infectious periods respectively. We simulated the dynamics using the Euler-Maruyama numerical scheme with the noise term corresponding to a stochastic transmission rate, which we modelled as standard geometric Brownian motion. The resulting system of stochastic difference equations are described schematically in Figure 1 in the main text and are given explicitly below:

Model compartments for individuals in Wuhan:

$$S(t+1) = S(t) - \beta(t)\, S(t)\, [\, I_{1w}(t) + I_{2w}(t)\,]/N$$

$$E_{1w}(t+1) = E_{1w}(t) + (1 - f)\, \beta(t)\, S(t)\, [\, I_{1w}(t) + I_{2w}(t)\,]/N - 2\sigma\, E_{1w}(t)$$

$$E_{2w}(t+1) = E_{2w}(t) + 2\sigma\, E_{1w}(t) - 2\sigma\, E_{2w}(t)$$

$$I_{1w}(t+1) = I_{1w}(t) + 2\sigma\, E_{2w}(t) - 2\gamma\, I_{1w}(t)$$

$$I_{2w}(t+1) = I_{2w}(t) + 2\gamma\, I_{1w}(t) - 2\gamma\, I_{2w}(t)$$

$$Q_w(t+1) = Q_w(t) + 2\sigma\, E_{2w}(t)\, e^{-\gamma\kappa} - \kappa\, Q_w(t)$$

$$D_w(t+1) = D_w(t) + 2\sigma\, E_{2w}(t)\, e^{-\gamma\kappa}$$

$$C_w(t+1) = C_w(t) + \kappa\, Q_w(t)$$

Here $S(t)$ is the number of individuals in Wuhan susceptible at time t; $E_{1w}(t)$ and $E_{2w}(t)$ are individuals in Wuhan the first and second period of their Erlang distributed incubation period respectively; $I_{1w}(t)$ and $I_{2w}(t)$ are individuals in Wuhan in the first and second period of their Erlang distributed infectious period respectively; $Q_w(t)$ is the number of symptomatic cases in Wuhan yet to be reported at time t; $D_w(t)$ is the

cumulative number of cases with symptoms in Wuhan at time *t*; $C_w(t)$ is the cumulative number of confirmed cases in Wuhan at time *t*. We include two compartments for I and E because the combination of these compartments with exponentially distributed waiting times creates an Erlang distribution with shape parameter 2, which is a more realistic representation of the peaked distribution of incubation and infectious period for 2019-nCoV (Figure S1).

Here $\beta(t)$ is the transmission rate at time *t*; σ is the rate of becoming symptomatic (i.e. 1/incubation period); γ = rate of isolation (i.e. 1/delay from onset-to-hospitalisation); κ is rate of reporting (i.e. 1/delay from onset-to-confirmation); *f* is the fraction of cases that travel; *N* is the population size in Wuhan. We therefore implicitly assume that all individuals become symptomatic, and this happens at the same time as they become infectious. The $e^{-\gamma\kappa}$ term in the equation for $Q_w(t)$ accounts for the probability that some cases may recover before being confirmed. A full parameter description is provided in Table S1.

Model compartments for traveller cases from Wuhan:

$$E_{1T}(t+1)= E_{1T}(t) + f\beta(t)\, S(t)\, [\, I_{1w}(t) + I_{2w}(t)\,] - 2\sigma\, E_{1T}(t)$$

$$E_{2T}(t+1)= E_{2T}(t) + 2\sigma\, E_{1T}(t) - 2\sigma\, E_{2T}(t)$$

$$I_{1T}(t+1)= I_{1T}(t) + 2\sigma\, E_{2T}(t) - 2\gamma\, I_{1T}(t)$$

$$I_{2T}(t+1)= I_{2T}(t) + 2\gamma\, I_{1T}(t) - 2\gamma\, I_{2T}(t)$$

$$Q_T(t+1)= Q_T(t) + 2\sigma\, E_{2T}(t)\, e^{-\gamma\kappa} - \kappa\, Q_T(t)$$

$$D_T(t+1)= D_T(t) + 2\sigma\, E_{2T}(t)\, e^{-\gamma\kappa}$$

$$C_T(t+1)= C_T(t) + \kappa\, Q_T(t)$$

Here $E_{1T}(t)$ and $E_{2T}(t)$ are individuals who have travelled from Wuhan and who are in the first and second period of their Erlang distributed incubation period respectively; $I_{1T}(t)$ and $I_{2T}(t)$ are individuals who have travelled from Wuhan and who are in the first and second period of their Erlang distributed infectious period respectively; $Q_T(t)$ is the number of symptomatic cases among travellers from Wuhan yet to be reported at time *t*; $D_T(t)$ is the cumulative number of cases among travellers from Wuhan with symptoms at time t; $C_T(t)$ is the cumulative number of confirmed cases among travellers from Wuhan at time *t*.

Transmission is modelled as geometric Brownian motion:

$$d \log(\beta(t)) = a\, dB_t$$

where *a* is the volatility of transmission over time and B_t is Brownian motion. As the Euler-Maruyama algorithm works in discrete time, at each step, we implement this geometric Brownian motion by sampling a random normally distributed number *X* with mean 0 and standard deviation *a*, then define $\beta(t+1) = \beta(t)e^X$.

Internationally exported cases

Cases that travelled internationally and became symptomatic, $D_T(t)$, and were later confirmed, $C_T(t)$, were distributed among other countries based on proportional risk inferred from connectivity to those countries (1,2). For example, if a country had a relative risk of export *W(country)* from Wuhan, then we would expect there to be $D_T(t)$ *W(country)* new symptomatic cases in this country at time *t*. We assumed no travel out of Wuhan occurred after 23rd January 2020, when extensive restrictions were put in place in Wuhan.

Model fitting

We estimated the time-varying transmission rate, $\beta(t)$, using sequential Monte Carlo (SMC) by jointly fitting to three datasets, with one used for validation:

1. Daily number of exported new cases from Wuhan (or lack thereof) in countries with high connectivity to Wuhan (i.e. top 20 most at risk), by date of onset. We only consider onsets up to 26th January 2020, as many of the cases detected after this point were not travellers from Wuhan (3).

2. Daily number of new cases in Wuhan (subsequently confirmed) with no market exposure, by date of onset, between 1st December 2019 and 1st January 2020 (4). This is combined with daily number of new cases in China, by date of onset (also subsequently confirmed), between 29th December 2019 and 23rd January 2020, which we assume that these are all in Wuhan (5). The most recent five data points from the China data were omitted during fitting as they were likely to be strongly influenced by delays in reporting.

3. Prevalence of infection on evacuation flights from Wuhan (see Table S3 for more details).

4. Validation dataset (not used for fitting): Data on confirmed cases in Wuhan from 16th January to 12th February (6)

5. Validation dataset (not used for fitting): Daily number of new exported infection from Wuhan (or lack thereof) in countries with high connectivity to Wuhan (i.e. top 20 most at risk), by date of confirmation, up to 10th February (7). We only considered individual export events (i.e. a family of travellers was counted as a single export).

To calculate the likelihood, we first used the model outputs to calculate expected values for each of the datasets we were fitting to:

1. International onset data was not disaggregated by country and so we used the total daily exported cases from Wuhan as our expected value, scaled by the proportion that would have detectable symptoms and proportion with known onsets: $D_T(t)\ \omega\ \rho_T$.

2. The number of new onsets per unit time reported in Wuhan was given the number of new onsets in the model, scaled by the relative reporting probability within Wuhan, the proportion that were detectable, and the proportion of confirmed cases with known onset: $D_w(t)\ \omega\ \delta\ \rho_w$.

3. The number of new confirmed cases in Wuhan was given by the number of new confirmed cases in the model, scaled by the proportion that were detectable and relative reporting probability within Wuhan $C_w(t)\ \omega\ \delta$.

4. The proportion of positive evacuated cases was assumed to be equivalent to the proportion of infectious individuals in Wuhan that would not be detected by thorough symptom screening at departure and were infected sufficiently long ago to have a positive test on arrival (as it takes time to test positive (8)). We therefore assumed infected individuals in Wuhan in the second half of their incubation period would be detectable at arrival, either through testing or subsequent appearance of symptoms during travel. This fraction is equivalent to: $[E_{2w}(t) + (1-\omega)\ (I_{1w}(t) + I_{2w}(t))\ ] / N$.

5. As destination country was known for confirmed exported cases, we generated 20 timeseries for cases exported (or not) to most at-risk countries each day (see Table S2). Hence we would expect $C_T(t)$ *W(country)* cases to be confirmed in a specific country at time t; the total number of confirmed cases to date is therefore the sum of these values up to the date being considered.

Here ω is the proportion of cases that would be eventually have detecteable symptoms, ρ_w and ρ_T are the proportion of confirmed cases in Wuhan and exported from Wuhan that have known onsets and is δ the relative proportion of cases that are reported in Wuhan compared to internationally. We used a Poisson observation model fitted jointly to datasets 1–3, using the expected values from model outputs 1–3 above.

The time-varying R_t was defined by $\beta(t)/\gamma$. Estimates for $\beta(t)$ were generated by running 200 repetitions of SMC with 2000 particles. The transmission volatility (a) and relative reporting of cases inside Wuhan (δ) were selected based on a grid search. Profile likelihoods for each parameter were constructed based on the joint likelihood distribution, with splines fitted to the estimates to calculate the MLE and 95% CI, defined as the point at which the spline crossed a line 1.92 units below the MLE (Figure S2–S4). Results shown in the main text use the MLE. We assumed the outbreak started on 22nd November with 1 infectious individual (9) and the population was initially fully susceptible. We assumed that the population in Wuhan was 11m. We also assumed all internationally exported infections would be eventually be detected in destination country if they travelled by plane.

Branching process simulation model
We used a branching process with a negative binomial offspring distribution (10, 11) to calculate the probability P that an outbreak starting with a single imported case would fail to go extinct (i.e. would cause a large outbreak). We also calculated the probability that an outbreak would occur after n introductions: $1-(1-P)^n$. We used the following representative parameter values in our analysis: 0.16 (95% CI: 0.11–0.64) for SARS-CoV and 0.25 (95% CI: 0.09–0.91) for MERS-CoV (12), and 0.54 (95% CI: 0.014 – ∞) for SARS-CoV-2 (13). We have built a Shiny online tool so these results can be explored further:
https://cmmid-lshtm.shinyapps.io/new_outbreak_probability/

Data and code availability
All data and code required to reproduce the analysis is available at:
https://github.com/adamkucharski/2020-ncov/stoch_model_V2_paper

Sensitivity analysis
To check the robustness of our results, we repeated our analysis with a larger assumed initial number of cases (i.e. 10 rather than 1), but this did not change our overall conclusions (Figure S5). As another sensitivity analysis, we used different flight data, taken from WorldPop rather than MOBS lab. However, again this did not change our overall conclusions (Figure S6). We also considered the effect of allowing individuals in the second half of their incubation period (i.e. E_2) to be infectious; this did not change our overall conclusions either (Figure S7).

**Parameter**	**Value**	**Distribution**	**Source**
Incubation period	5.2 days	Erlang (rate=2)	(15)
Infectious period	2.9 days	Erlang (rate=2)	(5)
Delay onset-to-confirmation	6.1 days	Exponential	(7)
Daily outbound passengers	3300		(14)
Population of Wuhan	11m		(14)
Fraction of cases that travel	3300/11m		
Initial cases	1		(9)
Introduction date	22nd November 2019		(9)
Proportion of Wuhan cases with onsets known (ρ_w)	0.16		(7)
Proportion of internationally exported cases with onsets known (ρ_T)	0.47		(3)
Relative reporting inside Wuhan compared to internationally (δ)	0.0066 (95% CI: 0.0029-0.009)		Fitted
Transmission volatility (a)	0.395 (95% CI: 0.138-0.693)		Fitted
Proportion of infections that are eventually detectable (ω)	1 (95% CI: 0.518-1)		Fitted

Table S1: Parameters used in the model, and assumed values.

Country	Relative risk
Thailand	0.1382
Japan	0.1334
Taiwan	0.1095
Korea	0.1094
USA	0.0533
Singapore	0.0483
Malaysia	0.0430
Vietnam	0.0358
Australia	0.0353
Philippines	0.0330
Indonesia	0.0303
Cambodia	0.0258
United Kingdom	0.0182
Canada	0.0151
Russian Federation	0.0127
Germany	0.0119
United Arab Emirates	0.0118
India	0.0113
Italy	0.0092
France	0.0088

Table S2: List at risk countries used in main analysis, and relatively probability of export to this country.

Destination (country)	Date	No. of passengers	Confirmed cases	Source
Singapore	30/01/2020	92	2	https://www.straitstimes.com/singapore/all-92-evacuated-from-wuhan-on-scoot-flight-being-tested
Belgium	02/02/2020	9	1	https://www.channelnewsasia.com/news/world/wuhan-coronavirus-virus-belgium-tests-positive-china-12394176
Korea	31/01/2020	368	1	https://www.cdc.go.kr/board/board.es?mid=a30402000000&bid=0030&act=view&list_no=365941&tag=&nPage=2
Malaysia	04/02/2020	107	2	https://www.channelnewsasia.com/news/asia/wuhan-coronavirus-malaysia-two-positive-evacuated-12396282
Japan	29/01/2020	206	4	https://www.mhlw.go.jp/stf/newpage_09199.html
Japan	30/01/2020	210	2	https://www.mhlw.go.jp/stf/newpage_09205.html
Japan	31/01/2020	149	4	https://www.mhlw.go.jp/stf/newpage_09278.html
Germany	01/02/2020	128	2	https://www.dw.com/en/coronavirus-german-evacuation-flight-lands-in-frankfurt/a-52223609
Japan	30/01/2020	206	3	https://www.reuters.com/article/china-health-japan/update-5-three-japanese-evacuees-from-wuhan-test-positive-for-virus-2-had-no-symptoms-idUSL4N29Z0CS
Italy	02/02/2020	56	1	https://www.thelocal.it/20200203/coronavirus-italians-evacuated-from-china-quarantined-rome

Table S3: Data on prevalence among evacuated individuals from Wuhan.

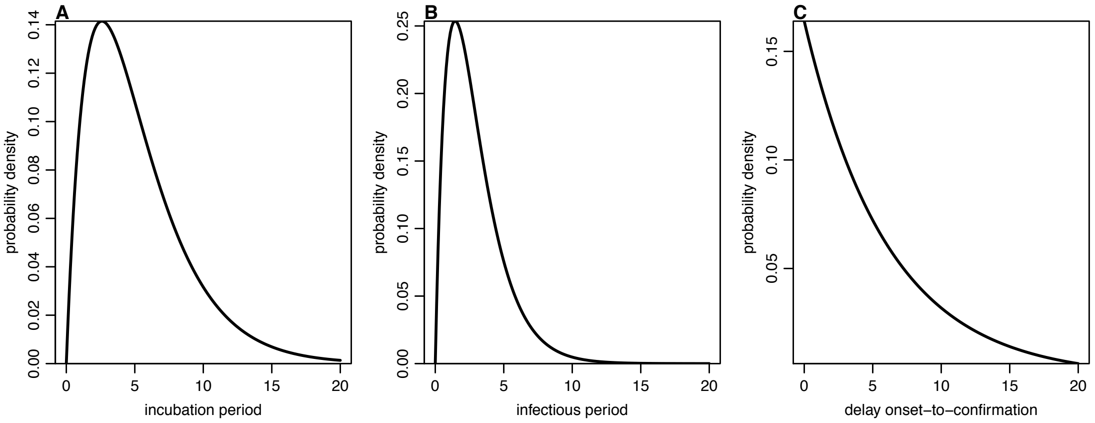

Figure S1: Assumed distribution of parameters. A) Erlang distributed incubation period. B) Erlang distributed infectious period. C) Exponentially distributed time from onset to report.

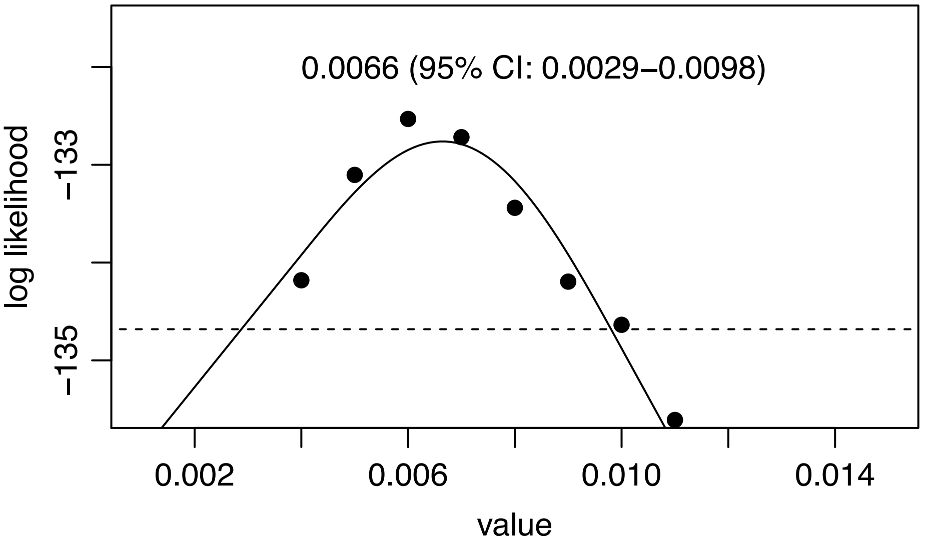

Figure S2: Profile likelihood for the relative reporting of confirmed cases within Wuhan compared to internationally exported cases (δ).

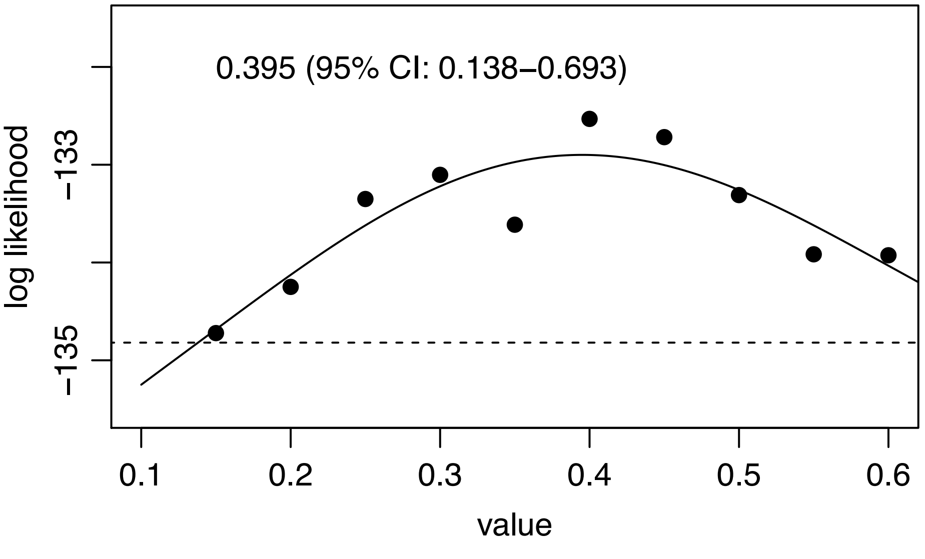

Figure S3: Profile likelihood for the transmission volatility parameter (a).

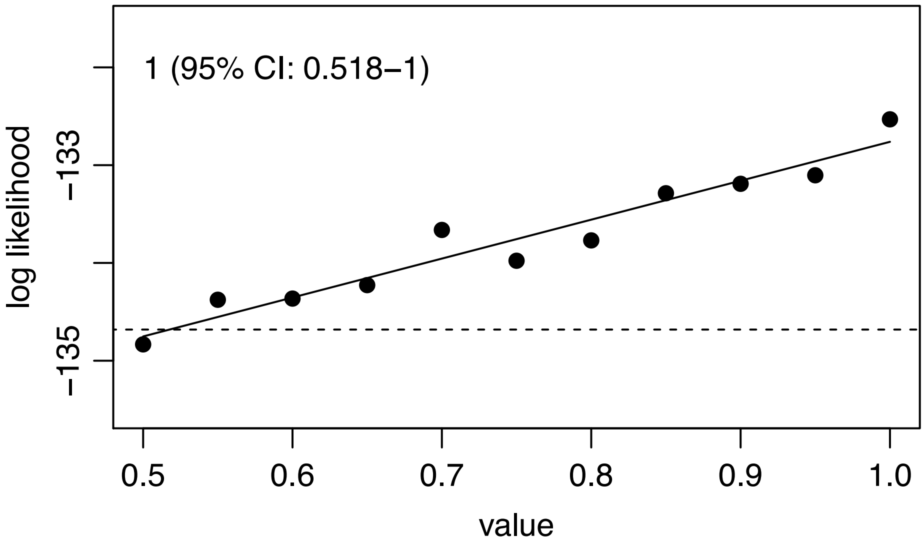

Figure S4: Profile likelihood for the proportion of cases that eventually have detectable symptoms (ω).

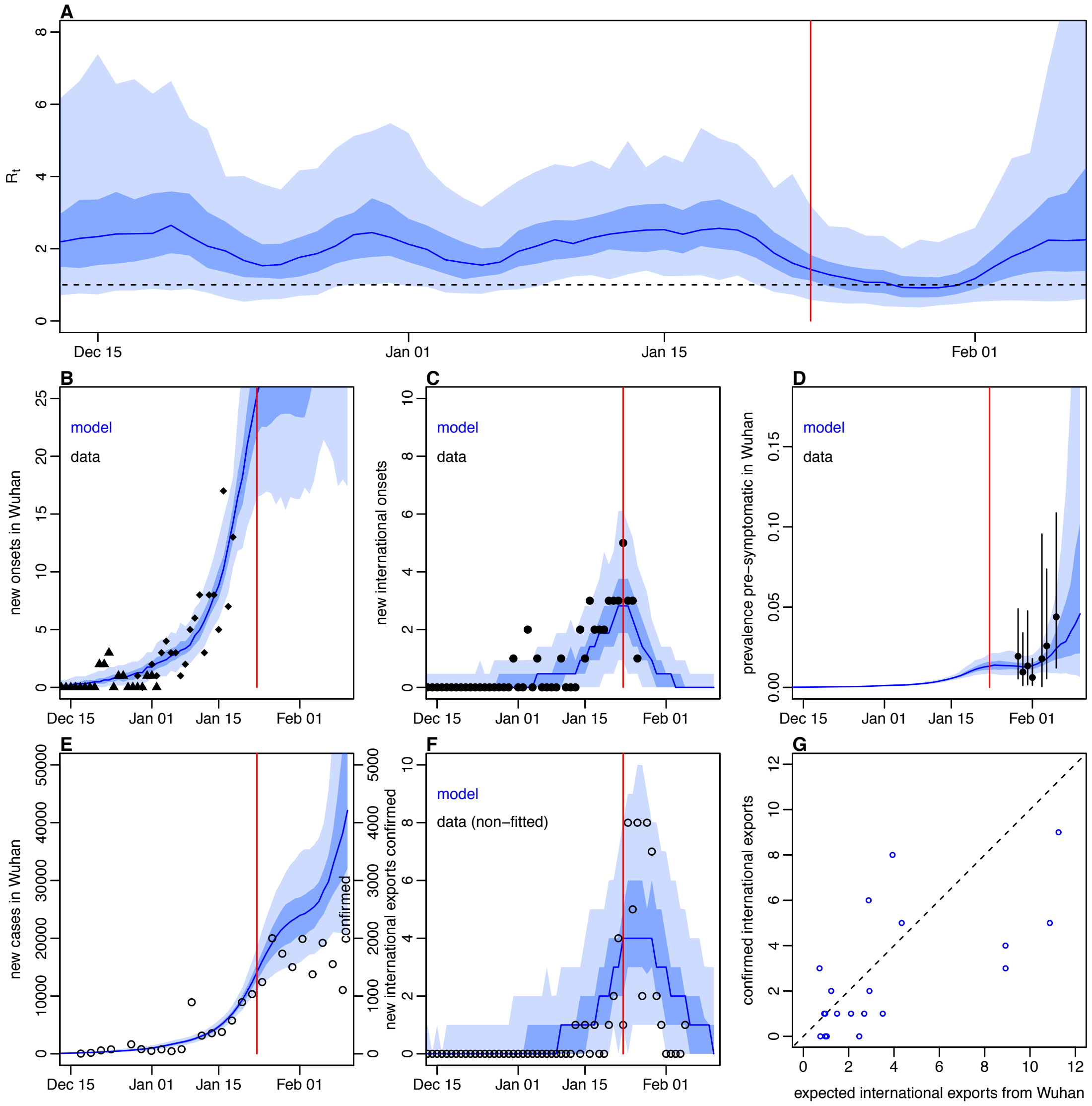

Figure S5: Model outputs when we assumed 10 initial cases rather than 1 on 22nd November 2019.

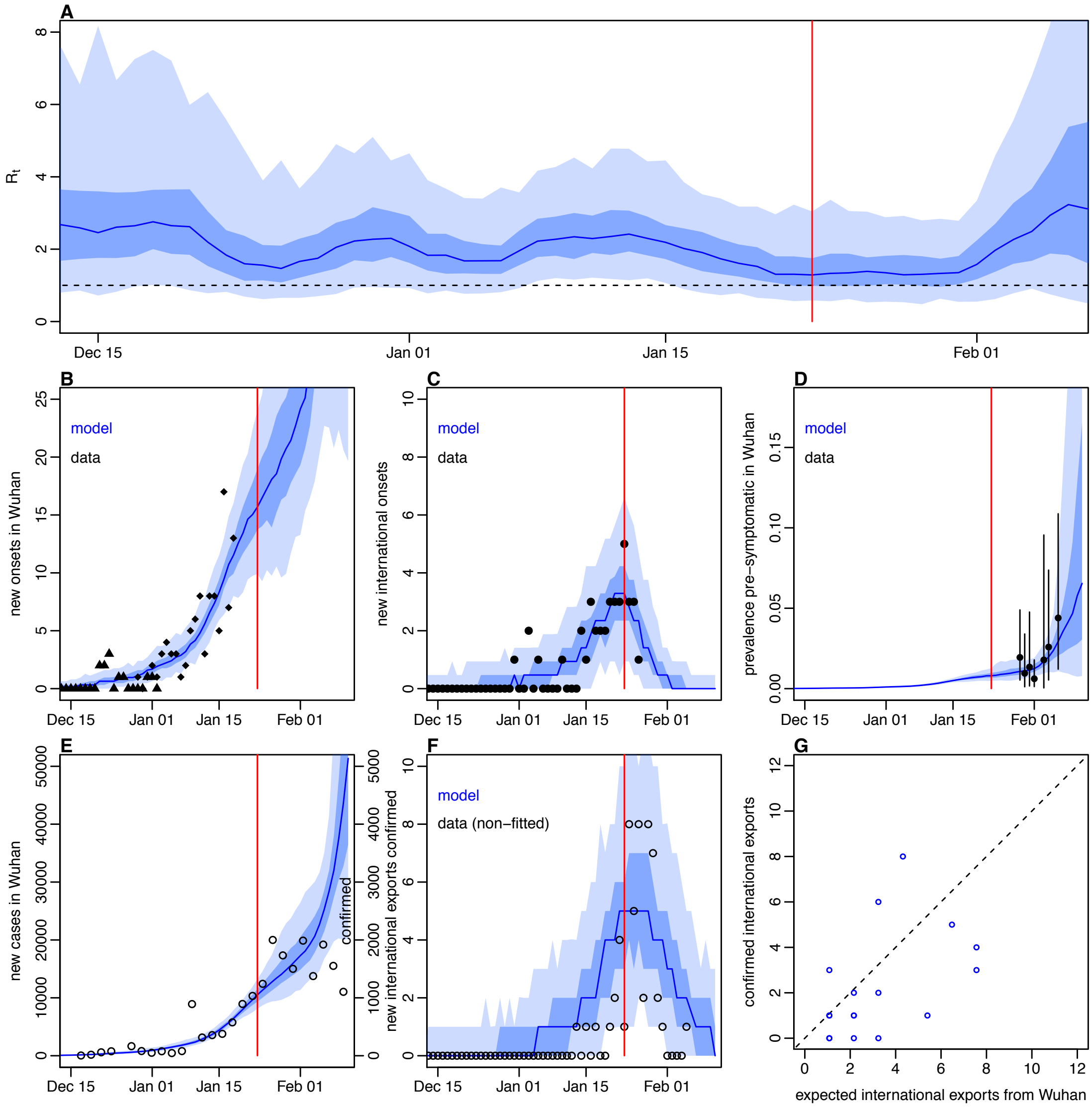

Figure S6: Model outputs when international traveller data from WorldPop is used instead of MOBS Lab estimates.

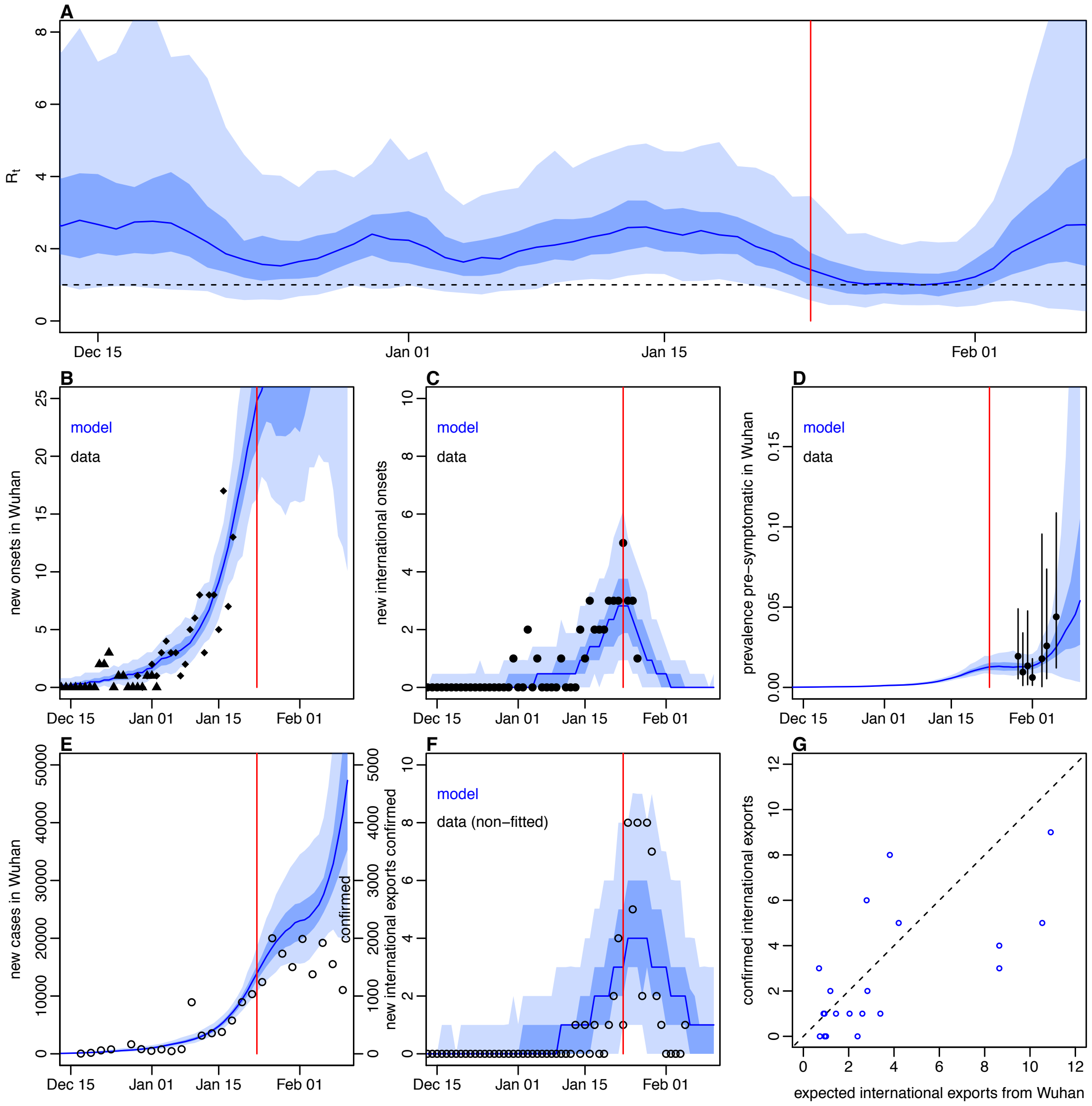

Figure S7: Model outputs when we assume infected individuals can transmit in the second half of their incubation period.

References

1. MOBS Lab. Situation Report Mainland China [Internet]. [cited 2020 Jan 31]. Available from: https://www.mobs-lab.org/2019ncov.html

2. Lai S, Bogoch II, Watts A, Khan K, Li Z, Tatem A. Preliminary risk analysis of 2019 novel coronavirus spread within and beyond China:20.

3. World Health Organisation. Coronavirus disease 2019 (COVID-19). Situation Report 24. WHO; 2020.

4. Huang C, Wang Y, Li X, Ren L, Zhao J, Hu Y, et al. Clinical features of patients infected with 2019 novel coronavirus in Wuhan, China. The Lancet. 2020 Jan;S0140673620301835.

5. Liu T, Hu J, Kang M, Lin L, Zhong H, Xiao J, et al. Transmission dynamics of 2019 novel coronavirus (2019-nCoV) [Internet]. Systems Biology; 2020 Jan [cited 2020 Jan 31]. Available from: http://biorxiv.org/lookup/doi/10.1101/2020.01.25.919787

6. National data statistics for COVID-19. Available from: https://ncov.dxy.cn/ncovh5/view/pneumonia

7. nCoV-2019 Data Working Group. Epidemiological Data from the nCoV-2019 Outbreak: Early Descriptions from Publicly Available Data. 2020.

8. World Health Organization. Use of laboratory methods for SARS diagnosis. Available from: https://www.who.int/csr/sars/labmethods/en/

9. Rambaut A. Phylodynamic Analysis | 44 genomes | 29 Jan 2020. Virological; 2020.

10. Lloyd-Smith JO, Schreiber SJ, Kopp PE, Getz WM. Superspreading and the effect of individual variation on disease emergence. Nature. 2005 Nov;438(7066):355–9.

11. Hellewell J, Abbott S, Gimma A, Bosse NI, Jarvis CI, Russell TW, Munday JD, Kucharski AJ, Edmunds WJ, CMMID nCoV working group, Funk S, Eggo RM. Medrxiv. 2020 February; doi: https://doi.org/10.1101/2020.02.08.20021162

12. Kucharski AJ, Althaus CL. The role of superspreading in Middle East respiratory syndrome coronavirus (MERS-CoV) transmission. Eurosurveillance [Internet]. 2015 Jun 25 [cited 2020 Jan 31];20(25). Available from: http://www.eurosurveillance.org/content/10.2807/1560-7917.ES2015.20.25.21167

13. Riou J, Althaus CL. Pattern of early human-to-human transmission of Wuhan 2019 novel coronavirus (2019-nCoV), December 2019 to January 2020. Eurosurveillance [Internet]. 2020 Jan 30 [cited 2020 Jan 31];25(4). Available from: https://www.eurosurveillance.org/content/10.2807/1560-7917.ES.2020.25.4.2000058

14. Imai N, Dorigatti I, Cori A, Donnelly C, Riley S, Ferguson NM. Report 2: Estimating the potential total number of novel Coronavirus cases in Wuhan City, China. 2020;6.

15. Li Q, Guan X, Wu P, Wang X, Zhou L, Tong Y, et al. Early Transmission Dynamics in Wuhan, China, of Novel Coronavirus–Infected Pneumonia. N Engl J Med. 2020 Jan 29;NEJMoa2001316.
